# A solitary pulmonary nodule caused by *Mycobacterium avium* with pleural effusion and pleuritis after transbronchial biopsy: a case report

**DOI:** 10.1186/s13256-021-02929-9

**Published:** 2021-07-11

**Authors:** Shigenari Nukaga, Hiroaki Murakami, Kazuma Yagi, Ryosuke Satomi, Takahiko Oyama, Arafumi Maeshima, Yoshitaka Oyamada

**Affiliations:** 1 Respiratory Medicine, National Hospital Organization Tokyo Medical Center, 2-5-1, Higashigaoka, Meguro, Tokyo 152-8902 Japan; 2 National Hospital Organization Tokyo Medical Center, Pathology, Japan

**Keywords:** *Mycobacterium avium* complex pulmonary disease, Solitary pulmonary nodule, Pleuritis, Pneumothorax

## Abstract

**Background:**

Pleural effusion and pleuritis are uncommon manifestations of *Mycobacterium avium* complex pulmonary disease. Pleuritis caused by *Mycobacterium avium* complex pulmonary disease presenting as a solitary pulmonary nodule is extremely rare. The pathogenesis of *Mycobacterium avium* complex pleuritis has not been elucidated. However, it has been suggested that secondary spontaneous pneumothorax from *Mycobacterium avium* complex pulmonary disease is one of the causes of *Mycobacterium avium* complex pleuritis.

**Case presentation:**

A 67-year-old Japanese woman who presented with a solitary pulmonary nodule developed a transient pneumothorax after transbronchial biopsy. A definitive diagnosis of solitary pulmonary nodule could not be made on bronchoscopy, so video-assisted thoracoscopic surgery was performed 1 month after bronchoscopy. On the day of hospitalization for the procedure, a left-sided pleural effusion appeared on a chest radiograph. Thickening of the parietal and visceral pleura and numerous scattered white small granules were seen on thoracoscopy. Histologic examination of the resected left lower lobe and a biopsy of the parietal pleura showed *Mycobacterium avium* complex solitary pulmonary nodule and *Mycobacterium avium* complex pleuritis.

**Conclusion:**

Iatrogenic pneumothorax can be a cause of pleuritis in a patient with *Mycobacterium avium* complex pulmonary disease. Clinicians should watch for the appearance of secondary pleuritis after transbronchial biopsy even in a patient with localized disease such as *Mycobacterium avium* complex solitary pulmonary nodule.

## Background

The incidence of pulmonary disease caused by nontuberculous mycobacteria (NTM) has been increasing globally [1, 2]. *Mycobacterium avium* complex (MAC), including *Mycobacterium avium* and *Mycobacterium intracellulare*, is the most common cause of NTM pulmonary disease (NTM-PD) [3, 4]. The radiologic findings of MAC pulmonary disease (MAC-PD) are commonly classified as fibrocavitary or nodular bronchiectatic [5]. However, some cases of MAC-PD present as a solitary pulmonary nodule (MAC-SPN) [6, 7]. Although MAC-PD generally shows chronic and indolent progression [7], pleuritis may occur as a rare complication [8–12]. The pathogenesis of MAC pleuritis has not been elucidated. However, it has been suggested that pneumothorax is one of the causes of MAC pleuritis [8]. In this article, we describe a patient with MAC-SPN who developed pleuritis after pneumothorax attributable to transbronchial biopsy.

## Case presentation

**Fig. 1:**
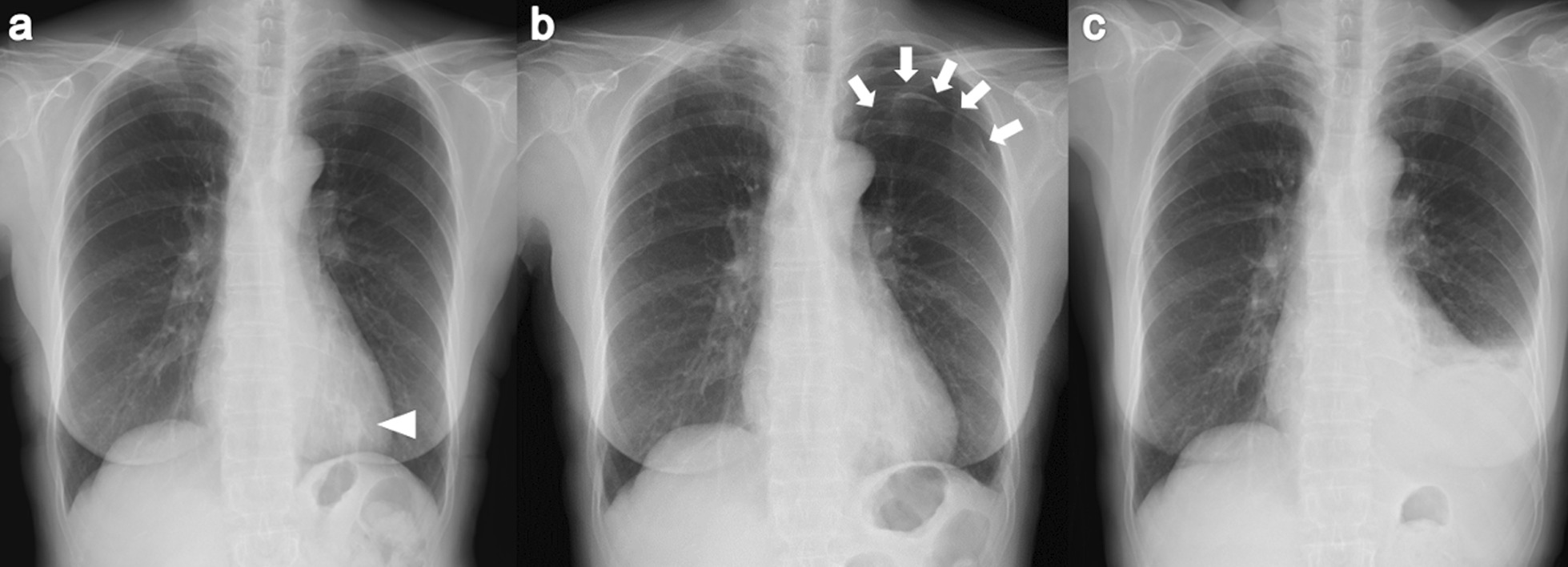
Chest radiograph of the lung. **a** Chest radiograph obtained before performing bronchoscopy showing a solitary nodule in the left lower lung field (arrowhead). **b** Chest radiograph obtained immediately after bronchoscopy showing a left-sided pneumothorax (arrow). **c** Chest radiograph obtained on the day of hospitalization for surgery showing a left-sided pleural effusion.

**Fig. 2:**
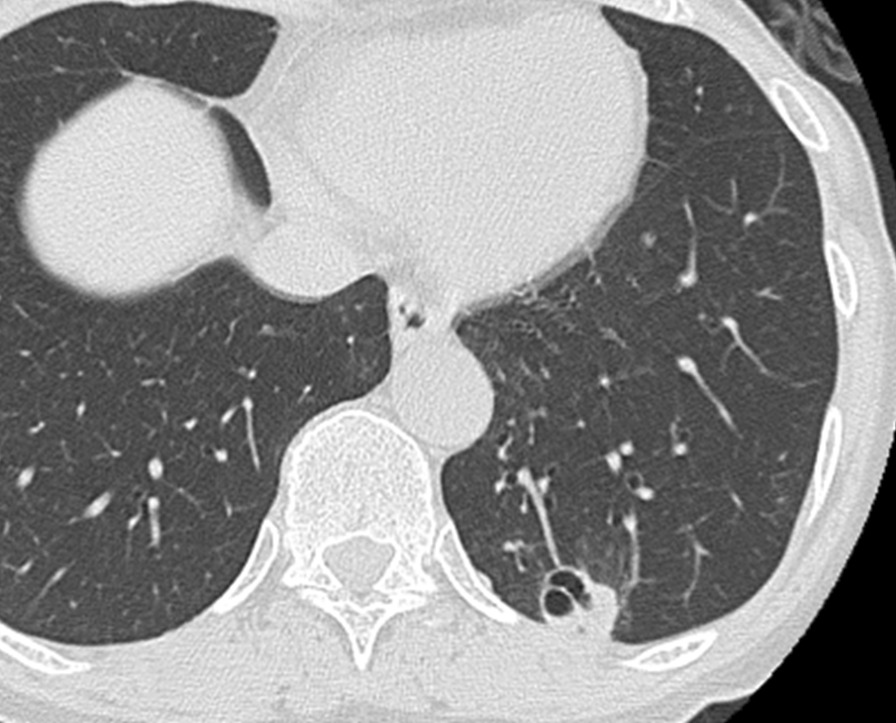
Computed tomography scan of the chest. A computed tomography scan of the chest showing an irregularly shaped solitary nodule in the periphery of the left lower lobe. The nodule was in contact with the pleura, and bronchial dilatation was seen inside the nodule

**Fig. 3:**
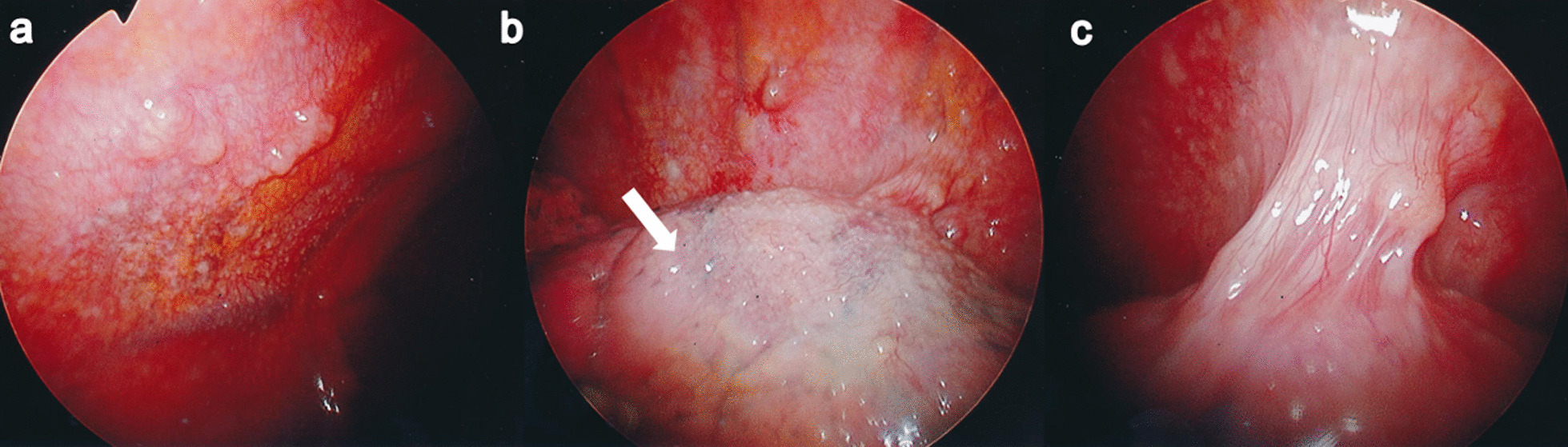
Thoracoscopic images. **a** Parietal pleura and **b** visceral pleura (arrow) were slightly thickened with numerous scattered white small granules. **c** Partial adhesion between the parietal and visceral pleura

**Fig. 4:**
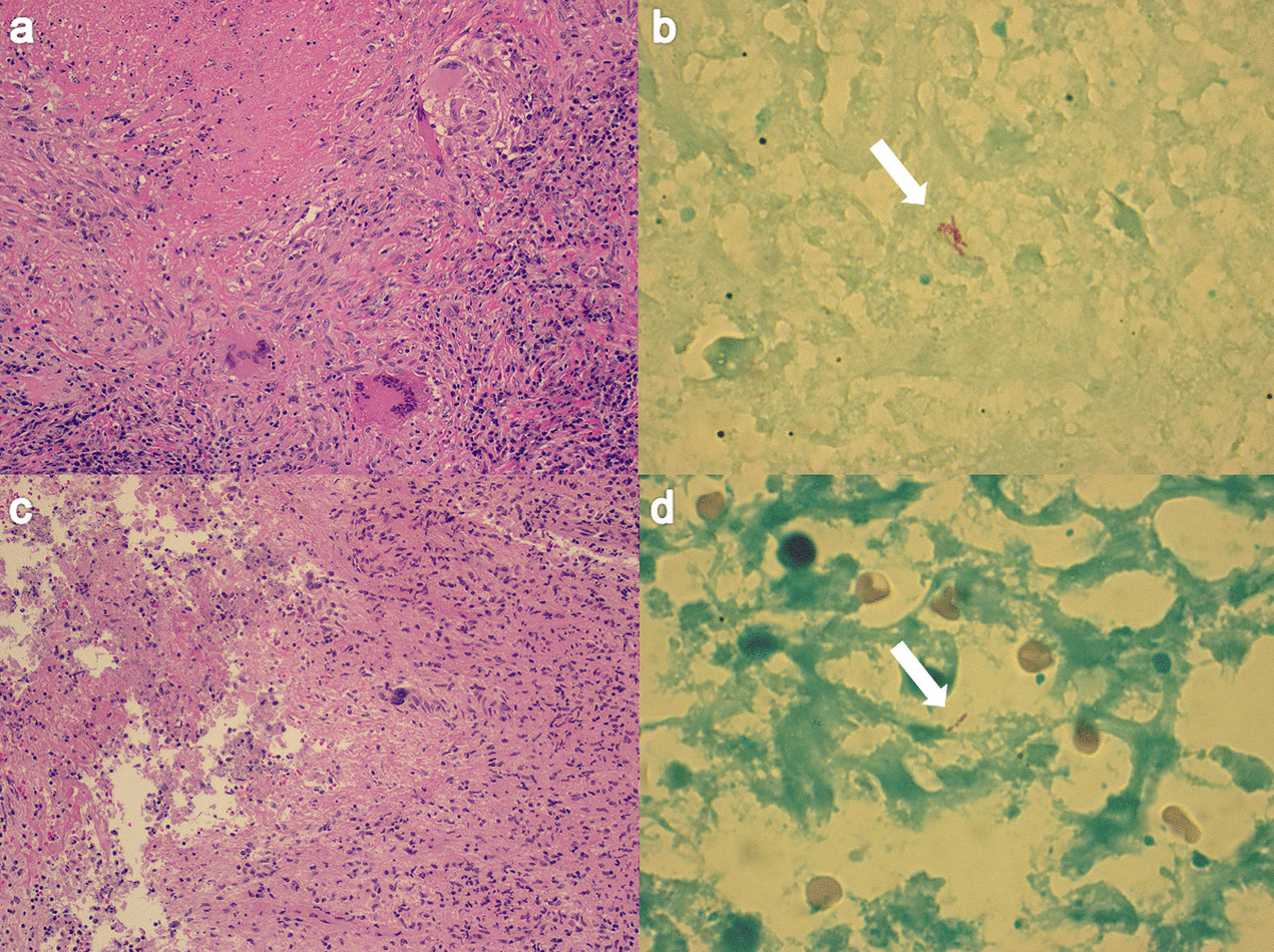
Histologic findings for the lung (**a**, **b**) and parietal pleura (**c**, **d**). Photomicrographs showing an epithelioid cell granuloma with caseous necrosis in the resected lung (**a**) and the parietal pleura (**c**) (hematoxylin and eosin stain, ×10). The photomicrographs show acid-fast bacilli in the resected lung (**b**) and parietal pleura (**d**) (arrows, Ziehl–Neelsen stain, ×10)

A 67-year-old Japanese woman was referred to our hospital for investigation of an abnormal shadow in the left lower lung field on chest radiography (Fig. 1a). She was asymptomatic and had no significant past medical history. Chest computed tomography (CT) showed a solitary nodule measuring 22 mm in diameter in the periphery of the left lower lobe (Fig. 2). The laboratory findings, including tumor markers, an interferon-gamma release assay (T-SPOT.TB, Oxford, Immunotec, Abingdon, UK), β-d-glucan, and serum cryptococcal antigen were negative. There were no findings suggestive of immunodeficiency or diabetes mellitus. A bronchoscopy with transbronchial biopsy and bronchial lavage were performed to diagnose the solitary pulmonary nodule. Adequate specimens were collected; however, a chest radiograph obtained immediately after a bronchoscopy showed a left-sided pneumothorax (Fig. 1b). The pneumothorax was small and asymptomatic, so the patient was observed without intervention and recovered within a few days. Although *Mycobacterium avium* was cultured from bronchial lavage fluid, histologic examination of the biopsy specimens showed nonspecific chronic inflammation with atypical B-cell proliferation. We could not make a definitive diagnosis of the nodule, and malignancy was unable to be ruled out. To investigate the nodule further, surgical lung resection was planned. The patient was hospitalized for surgery 1 month after her bronchoscopy. Although she had no fever, cough, or shortness of breath, a chest radiograph on the day of hospitalization showed a left-sided pleural effusion (Fig. 1c). Given that there was no remarkable change in her general condition, video-assisted thoracoscopic surgery (VATS) was performed as planned. About 300 ml of pale yellow transparent pleural effusion was found in the thoracic cavity and removed by suction. The parietal and visceral pleura near the nodule were slightly thickened with numerous scattered white small granules. (Fig. 3a, b). There was partial adhesion between the parietal and visceral pleura (Fig. 3c). Partial resection of the left lower lobe including the nodule and a parietal pleura biopsy were performed. Histologic examination of the resected left lower lobe and parietal pleura showed an epithelioid cell granuloma with caseous necrosis (Fig. 4a, c). There was no evidence of malignancy. Although tissue and pleural fluid cultures were negative, Ziehl–Neelsen stain-positive bacilli were observed in both lung and pleural tissues (Fig. 4b, d). Based on the histological findings, the patient was diagnosed with SPN and pleuritis caused by *Mycobacterium avium* infection. Combination chemotherapy of rifampin (450 mg/day), ethambutol (500 mg/day), and clarithromycin (800 mg/day) for *Mycobacterium avium* infection was initiated after surgery. She had no complications, and there was no recurrence of pleural effusion 6 months after initiating postoperative chemotherapy.

## Discussion and conclusions

Pleural effusion and pleuritis are uncommon manifestations of NTM infection [8–12]. Previous studies have reported that NTM pleuritis occurs in 1.2% of patients with NTM-PD [9], while MAC pleuritis occurs in 2.9–5.0% of patients with MAC-PD [9–11]. Although the radiologic findings of MAC-PD are classified commonly into two types, that is, a fibrocavitary form and a nodular bronchiectatic form [5], there have been a few cases of MAC-SPN [6, 7]. MAC pleuritis tends to be seen more frequently in the fibrocavitary form than in the nodular bronchiectatic form [8, 9]. However, pleuritis caused by MAC-SPN is extremely rare. To our knowledge, there is only one report of pleuritis attributable to MAC-SPN [13], and our case is the first report of MAC-SPN with pleuritis showing pleural effusion.

The mechanism via which pleuritis develops from NTM-PD has not been elucidated. However, it has been suggested that NTM may gain entry to the pleural cavity through a transient bacteremia or by direct spread from a subpleural lesion [14]. Moreover, two mechanisms have been proposed for direct spread. The first is direct infiltration of the infectious pulmonary lesion, and the second is leakage of NTM from the subpleural lesion into the pleural cavity through rupture of the pleura [8, 15].

It has been reported that secondary spontaneous pneumothorax (SSP) occurs in advanced stages of NTM-PD [16]. It has also been reported that SSP is a frequent complication of NTM pleuritis. In previous studies, pneumothorax was observed in 11 of 12, 2 of 9, and 5 of 11 cases of NTM pleuritis [8, 10, 17]. Hence, it has been suggested that pneumothorax is one of the causes of NTM pleuritis [8]. In our case, rapid development of pleural effusion after pneumothorax was observed. Furthermore, spontaneous pleuritis caused by MAC-SPN is extremely rare. Therefore, it is possible that pleural rupture arising from pneumothorax was the cause of the pleuritis.

However, unlike in the reported cases, the pneumothorax in our case was not secondary spontaneous but iatrogenic pneumothorax. Although SPNs can be caused by various disorders, including neoplasms, infection, inflammation, and vascular and congenital abnormalities [18], diagnosis of this disorder is often challenging. Clinicians perform invasive procedures, including bronchoscopic biopsies and image-guided transthoracic needle aspiration (TTNA), to make a pathologic diagnosis [19]. The incidence rates of pneumothorax diagnosed by bronchoscopic biopsy, CT-guided TTNA, and ultrasound-guided TTNA have been reported to be 0–5.1%, 20.5%, and 4.4%, respectively [20, 21]. A diagnosis of SPN involves a risk of pneumothorax.

Moreover, SPNs attributable to NTM (NTM-SPN) are often located immediately beneath the pleura and are in contact with the surface of the visceral pleura. Ose *et al*. reported that 85.7% of NTM-SPNs were subpleural lesion, and 55.6% were in contact with the pleura [22]. Therefore, leakage of NTM into the pleural cavity may occur easily when pneumothorax occurs in patients with NTM-SPN.

In addition, although surgical resection is considered curative in patients with MAC-SPN, the need for postoperative antibiotic treatment remains controversial. The guidelines state that antibiotic treatment is not usually required following resection in the absence of other features of MAC-PD [7, 23]. However, in patients with MAC-SPN who develop pneumothorax, leakage of MAC from the subpleural lesion into the pleural cavity may occur. Therefore, in such cases, it is necessary to carefully observe the pleura during surgery and consider antibiotic treatment following surgery if needed.

In conclusion, we have encountered a patient with MAC-SPN who developed pleuritis after pneumothorax subsequent to transbronchial biopsy. Although a causal relationship between MAC pleuritis and SSP has been suggested, iatrogenic pneumothorax can also be a cause of pleuritis attributable to NTM. Clinicians should watch carefully for the appearance of secondary pleuritis after transbronchial biopsy even in patients with localized disease such as NTM-SPN.

## Data Availability

Not applicable.

## Abbreviations

NTM: Nontuberculous mycobacteria
MAC: *Mycobacterium avium* complex
NTM-PD: NTM pulmonary disease
MAC-PD: MAC pulmonary disease
MAC-SPN: MAC-PD presenting as a solitary pulmonary nodule
CT: Computed tomography
VATS: Video-assisted thoracoscopic surgery
SSP: Secondary spontaneous pneumothorax
TTNA: Transthoracic needle aspiration
NTM-SPN: SPN caused by NTM
